# A Case of Severe Purulent Inflammation of the Middle Ear, with Restoration of the Drumhead; Consecutive Dentalgia without Caries

**Published:** 1883-08

**Authors:** Edward S. Peck

**Affiliations:** Ophthalmic and Aural Surgeon to Charity Hospital, New York; 53 West Fiftieth Street, New York City


### A CASE OF SEVERE PURULENT INFLAMMATION OF THE MIDDLE EAR, WITH RESTORATION OF THE DRUMHEAD; CONSECUTIVE DENTALGIA WITHOUT CARIES.

BY EDWARD S. PECK, M.A., M.D., OPHTHALMIC AND AURAL SURGEON TO CHARITY HOSPITAL, NEW YORK.

The accompanying case is published for the following considerations:

1st. As an example of restoration of the ear-drum after a total loss of the same, together with a return of hearing.

2nd. To illustrate the anatomical connection between dentalgia and severe purulent otitis media.

Mr. C., formerly known in the west as the builder of an opera house, was attacked in June, 1880, with a severe purulent inflammation of the right middle ear; the left ear was partially deaf from chronic tympanic catarrh. The purulent otitis (right) was caused by exposure to cold, and was purely consecutive, having followed an acute naso-pharyngitis of ten days' standing. My note-book gives the following statistics: June 6th, auditory canal and middle aural region of right side very painful; canal filled with white, thick pus; on cleansing with cotton there was a complete perforation of the membrana tympani, only a narrow rim being attached to the annulus tympanicus, while the hammer was displaced upwards and backwards nearly parallel with the superior wall of the tympanum. A loud-ticking test-watch, capable of being heard twenty-four feet in a quiet room, could be heard only on firm contact with the ear. The treatment consisted in thorough cleansing with the syringe, hot boracic water, and a solution of sulpho-carbolate of zinc, while a continuous douche of hot water was used to control pain. To the anterior and posterior nares, which were still congested and bathed in muco-pus, a zinc spray was thoroughly used. This regime was continued nearly one month, until on July 2d, the hearing distance on the right side was one-half an inch. The Politzer and dry methods of inflating the middle ears had been regularly used, and the hearing distance in the left ear, previously fifteen inches, had increased to three feet. At this date the aural discharge was nearly stopped. The severe symptoms of pain and tinnitus had disappeared. July 10th, or eight days later, every symptom was less severe; the aural discharge had ceased; the nasal discharge was slight; the perforation was partially covered with new fibrous tissue, and the hearing distance had increased to eight inches by the watch.

No further record of the patient's symptoms was made, as he went regularly to business, and passed from my hands. On November 7th, of the same year, the perforation of the right membrana tympani was entirely healed, with a restored position of the hammer, though not of the light spot, while the new membrane was movable to inflation by the method of Volsalva. The hearing distance in each ear was *five feet* by the watch, and *twenty feet* to a clear whisper. It is a well-known fact to the aurist that there is often a disparity between tests of hearing by the watch and human voice in whisper, the better results accruing to the second method, while the watch is a more accurate test. The voice is the habitual criterion for the deaf person, whether in whisper or intensified to various degrees.

From the second week of the aural complication until the cessation of discharge and abatement of pain, this patient suffered from toothache referable to the two last molars. No caries could be found, and the teeth themselves were not sensitive to the touch of a steel instrument. The dentalgia was constant, and at times severe.

The study of otalgia associated with dental irritation is an interesting one. Not counting the large class of cases of dental caries, where this factor is the manifest cause of otalgia, examples of consecutive dentalgia with antecedent aural disease of severity are of paramount interest. The explanation is purely anatomical, and is as follows: of the three divisions of the fifth pair of cranial nerves, the inferior maxillary, through its inferior dental branch, is the only one which supplies the teeth of the lower jaw; this nerve is mixed in character, its nerves of sensation passing into each tooth to the pulp, through a perforation in each individual fang. With the fifth nerve are connected four ganglia, of which the otic is connected with the inferior maxillary division, and is the only one with which we are here concerned. The otic ganglion is situated on the inner surface of the inferior maxillary nerve, near its projection from the parent nerve. It sends branches of communication to the inferior maxillary between the two pterygoid muscles, fibres of which communicating branches are carried on to the inferior dental nerve. It also receives branches from the glosso-pharyngeal, a division of the eighth pair, through the small superficial petrosal nerve, which is continued from the tympanic pexus of the middle ear. In this way the nerve supply of the tympanic cavity is connected with the sensory fibres of the inferior dental by the intervention of the otic ganglion.

53 West Fiftieth Street, New York City.

